# Optimizing teacher basic need satisfaction in distributed healthcare contexts

**DOI:** 10.1007/s10459-021-10061-y

**Published:** 2021-07-04

**Authors:** M. J. M. Verhees, R. E. Engbers, A. M. Landstra, G. A. M. Bouwmans, J. J. Koksma, R. F. J. M. Laan

**Affiliations:** 1 Radboudumc Health Academy, Radboudumc, Gerard van Swietenlaan 2, 6525 GB Nijmegen, The Netherlands; 2 Rijnstate, Arnhem, The Netherlands

**Keywords:** Context, Distributed medical education, District teaching hospital, Primary care, Teacher motivation, Self-determination theory, University hospital

## Abstract

Optimizing teacher motivation in distributed learning environments is paramount to ensure high-quality education, as medical education is increasingly becoming the responsibility of a larger variety of healthcare contexts. This study aims to explore teaching-related basic need satisfaction, e.g. teachers’ feelings of autonomy, competence and relatedness in teaching, in different healthcare contexts and to provide insight into its relation to contextual factors. We distributed a digital survey among healthcare professionals in university hospitals (UH), district teaching hospitals (DTH), and primary care (PC). We used the Teaching-related Basic Need Satisfaction scale, based on the Self-Determination theory, to measure teachers’ basic needs satisfaction in teaching. We studied relations between basic need satisfaction and perceived presence of contextual factors associated with teacher motivation drawn from the literature. Input from 1407 healthcare professionals was analyzed. PC healthcare professionals felt most autonomous, UH healthcare professionals felt most competent, and DTH healthcare professionals felt most related. Regardless of work context, teachers involved in educational design and who perceived more appreciation and developmental opportunities for teaching reported higher feelings of autonomy, competence, and relatedness in teaching, as did teachers who indicated that teaching was important at their job application. Perceived facilitators for teaching were associated with feeling more autonomous and related. These results can be utilized in a variety of healthcare contexts for improving teaching-related basic need satisfaction. Recommendations for practice include involving different healthcare professionals in educational development and coordination, forming communities of teachers across healthcare contexts, and addressing healthcare professionals’ intentions to be involved in education during job interviews.

## Introduction

Healthcare systems worldwide are evolving to keep up with the challenges of ensuring accessibility, affordability and quality of care, against the background of increasing clinical complexity that comes with an ageing population (Frenk et al., 2010; Koksma & Kremer, 2019; World Health Organization, 2017). To face these challenges, the focus in healthcare landscapes is shifting towards decentralized or distributed healthcare. Care locations are shifting closer to patients’ homes, and innovative healthcare contexts are emerging to facilitate, for instance, eHealth, highly specialized care, and multidisciplinary care (Araujo de Carvalho et al., 2017; Hardin & Mason, 2019; Schreiweis et al., 2019; Van Eenoo et al., 2018).

Decentralized healthcare also implies the redistribution of educational responsibilities between contexts such as university hospitals, district hospitals, healthcare facilities in primary care and in the public health sector, and possibly new healthcare contexts (van Schalkwyk et al., 2020a, 2020b; Van Schalkwyk et al., 2020a, 2020b). This is necessary to better align medical education, community healthcare needs and the future healthcare workforce. Learning environments distant from the academic hospital offer opportunities for different content in learning, such as continuity of care, social determinants of health and managing practice (de Villiers et al., 2017; Topps et al., 2015). Further, research indicates that trainees are more likely to pursue careers in community-based healthcare service if they were trained in them (de Villiers et al., 2017).

When healthcare professionals increasingly learn in a large variety of distributed healthcare environments, what we need is insight into factors that benefit medical educational quality in different contexts. More specifically, we need to enhance our understanding of factors relating to teacher quality as a wider range of healthcare professionals will be involved in teaching and educational quality is strongly determined by teachers' quality and their working conditions (Han & Yin, 2016; Royal, 2017; Zelek & Goertzen, 2018).

Teacher motivation is a valid measure to assess teacher quality as it is related to beneficial outcomes such as better teacher performance and well-being, and better student learning outcomes, motivation, and well-being (Bakker, 2005; Orsini et al., 2020; Pelletier et al., 2002; Roth et al., 2007). Although studies describe different contextual factors associated with teacher motivation, most do not show how exactly these factors relate to motivation, and consequently not how they can be used to enhance teacher motivation (Bartle & Thistlethwaite, 2014; Browne et al., 2018; Cochran Ward et al., 2013; Dahlstrom et al., 2005; DaRosa et al., 2011; Dybowski & Harendza, 2014; Engbers et al., 2015; Berg et al., 2013; J. W. van den Berg et al., 2015). In addition, these studies mostly predate the emergence of decentralized healthcare, and their single-curriculum and single-institute character, moreover, does not serve to promote the idea that the outcomes should serve a variety of healthcare contexts.

Our study aims to fill this gap by providing insight into how contextual factors relate to the teacher motivation of healthcare professionals working in different healthcare contexts. To measure teacher motivation, we used a questionnaire based on the Self-Determination Theory (SDT), a current theory on motivation that takes the influence of socio-contextual factors into account (Deci & Ryan, 2002). SDT distinguishes between more autonomous and controlled forms of motivation. SDT states that people’s motivation for an activity, such as medical teaching, can become more autonomous if their basic psychological needs of feeling autonomous, competent, and related to others are satisfied more (Gagne & Deci, 2005). Using these basic needs to break down the construct of motivation allows us to explain in more detail how contextual factors can support a more autonomous teacher motivation.
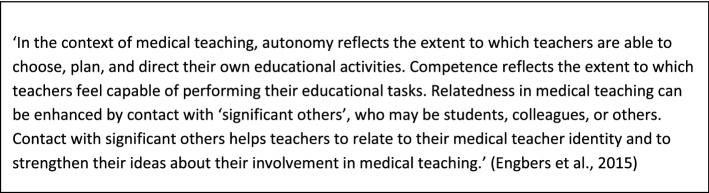


In short: we aim to provide insight into how contextual factors can be used to benefit teacher motivation in different healthcare contexts*.* To do so, we examined relations between teaching-related basic need satisfaction (e.g. feelings of autonomy, competence and relatedness in teaching, see Box 1) and the perceived presence of different contextual factors in the work environment that we know from literature to be associated with teacher motivation. We hypothesized that the perceived presence of recognition for teaching tasks, facilities for teaching, and opportunities for teacher development are positively associated with teachers’ feelings of autonomy, competence, and relatedness in medical teaching (Bartle & Thistlethwaite, 2014; Browne et al., 2018; Cochran Ward et al., 2013; Dahlstrom et al., 2005; DaRosa et al., 2011; Dybowski & Harendza, 2014; Engbers et al., 2015; Berg et al., 2013; Berg et al., 2015), and that the perceived presence of these factors differs between healthcare contexts, showing differences in teacher motivation. Insights into relations between teacher motivation and contextual factors can be used to assess and adjust both existing and newly emerging contexts offering distributed medical education, to improve teachers’ feelings of autonomy, competence, and relatedness in teaching, thus contributing to education quality.

## Methods

### Context

In the Netherlands, different healthcare settings are involved in medical education. University hospitals (UH) are involved in undergraduate, graduate and post-graduate medical education. They play a coordinating role for medical education in their own region, and are responsible for curriculum development and evaluation. Healthcare professionals working in UH are often involved in clinical education (in graduate and post-graduate education), and some also provide classroom education (in undergraduate and graduate education). District teaching hospitals (DTH) are affiliated to one or more university hospital. Healthcare professionals working in DTH are often involved in clinical training in graduate and post-graduate education. Some also provide clinical teaching to undergraduate medical students. Primary care facilities (PC) can provide clinical training in graduate and/or post-graduate medical education. Practitioners can voluntarily apply to be practitioner-educators, and are then affiliated to and supported by the primary care department of a UH.

### Participants and procedure

A digital survey was sent to all medical staff, physicians as well as specialist trainees and residents not in specialist training, in a university hospital and six affiliated district teaching hospitals in region East-Netherlands. To broaden the scope and extend the generalizability of the study, a second university hospital was recruited from region North and East Netherlands. The survey was also distributed among physicians working in primary care practices that have a teaching affiliation with the primary care department of the East Netherlands university hospital. Practices included general practitioners’ practices, elderly care facilities, and facilities for occupational medicine, insurance medicine, and addiction medicine.

A link to the web-based version of the survey, together with an information letter, was sent out to participants via email by the chairperson of the educational committee of each hospital/practice. The survey was administered anonymously. Data collection was conducted between June 2019 and March 2020. Informed consent was obtained from all participants prior to them filling out the questionnaire. This study received ethical approval from the Netherlands Society for Medical Education ethical review board (NVMO ERB, case no 333).

### Instrument

We used a digital survey consisting of four categories: (1) demographical information, (2) involvement in medical teaching, (3) teaching-related basic need satisfaction, and (4) contextual factors associated with motivation.

The first section contained questions on age, gender, working experience, and location of employment (institute)*.* Years of working experience measured the number of years the respondent had worked in their healthcare profession.

The second category contained questions regarding involvement in medical education. The percentage of time per week spent by teachers on teaching tasks was measured per 10 percent, and participants had to divide 100% of their time between patient care, education, research, and management. Teachers’ degree of participation in development of education and coordination of education was measured in four categories: participating not at all, a little, quite a lot, or a lot. The importance awarded by respondents to teaching tasks at the time of their job application at their current place of work was measured on a 4-point scale, ranging from not important (0) to very important (3).

The third category measured feelings of autonomy, competence, and relatedness in medical teaching. For this purpose, the Teaching-related Basic Need Satisfaction (T-BNS) questionnaire was integrated into the survey. The T-BNS is a self-report questionnaire, validated to measure motivation for teaching tasks in a study by Engbers et al. in a sample of medical teachers working in a university hospital (Engbers et al., 2015). The T-BNS measures motivation for teaching tasks using three subscales (feelings of autonomy, competence, and relatedness), consisting of six items each. Answers were given on a 5-point Likert Scale.

The fourth category addressed the perceived presence of different contextual factors. We performed a literature search with a broad scope to uncover contextual factors that are generally associated with teacher motivation (Bartle & Thistlethwaite, 2014; Browne et al., 2018; Cochran Ward et al., 2013; Dahlstrom et al., 2005; DaRosa et al., 2011; Dybowski & Harendza, 2014; Engbers et al., 2015; Berg et al., 2013; Berg et al., 2015). In an expert brainstorm, three main categories were recognized in these factors: recognition for the teacher role (5 items), facilitators for teaching (3 items), and opportunities for teacher development (5 items) (Table 1). The perceived presence of these factors was examined using a 5-point Likert scale.

**Table 1:** Contextual factors associated with teacher motivation

*Recognition*
When I perform teaching tasks, this is appreciated by my colleagues
When I perform teaching tasks, this is appreciated by the organization
In my workplace there are good role models in the domain of teaching
When I perform teaching tasks, this is appreciated by my patients
The learners I meet (students, trainee doctors, doctors not in specialist training, doctors in specialist training) have high motivation for learning
*Facilitators*
In my workplace there is enough time available for teaching tasks
In my workplace there is enough financial compensation available for teaching tasks
In my workplace there is enough organizational support available for teaching tasks
*Opportunities for teacher development*
In my workplace there is enough educational support available if required
In my workplace there are career opportunities in education and training
In my workplace there are enough learning activities available (refresher training) for teacher self-development
In my workplace I receive feedback on my teaching tasks
In my workplace I am free to choose in what learning activities (refresher training) in the domain of teaching I enroll

### Statistical analysis

Data were analyzed using SPSS (version 25). First, all items were examined with descriptive statistics.

Exploratory factor analysis was performed on T-BNS subscales and contextual factors associated with teacher motivation. For both scales, maximum likelihood factor analysis with Oblimin rotation was used, searching for three factors. Cronbach’s alpha was used to determine the internal consistency reliability. Subscale scores for the compound variables were calculated by taking the mean of the corresponding items, with higher scores equaling more basic need satisfaction or more perceived presence of contextual factors for T-BNS and questionnaire on contextual factors, respectively (scale 0–4).

Mean scores for feelings of autonomy, competence, and relatedness, and perceived presence of recognition for the teacher role, facilitators for teaching, and opportunities for teacher development were calculated for each healthcare context separately. Mean scores were compared using one-way ANOVA with post-hoc Bonferroni analysis, or with chi-square tests with post-hoc analysis using contingency tables with Bonferroni correction for percentages.

We subsequently assessed all variables that were possibly related to basic need satisfaction, using chi-square tests or Pearson correlation coefficient as appropriate, in order to identify the subset of variables that was most explanatory for feelings of autonomy, competence, and relatedness. Multiple regression was used to examine relations between teaching-related basic need satisfaction (feelings of autonomy, competence, and relatedness), and context of employment as well as contextual factors (recognition, facilitators, opportunities for teacher development, and involvement in coordination and development of education). Location of employment was recoded from institution names to groups of healthcare contexts: primary care (PC), district teaching hospital (DTH), and university hospital (UH). Healthcare professionals employed in a DTH as well as a UH were recoded into the UH group. We recoded the variable context of employment (UH, DTH, or PC) into dummy variables DTH and PC for the purpose of multiple regression analysis. Teachers’ degree of participation in development of education and coordination of education was recoded into two categories: participating not at all (0) and participating a little, quite a lot or a lot (1). We corrected for participants’ sex, years of working experience, proportion of teaching tasks in their total employment, and the importance they attached to education at the time of their job application.

## Results

### Respondents

Out of 6864 healthcare professionals who were invited to fill out the questionnaire, we received 2144 responses (response rate 31%). Ten respondents did not give informed consent, and 147 respondents were excluded because their position (dentists, researchers) did not match the target audience. In addition, 348 healthcare professionals reported that they were not involved in medical education and were excluded. An additional 232 respondents were excluded because they did not completely fill out the T-BNS questionnaire and/or the questionnaire on contextual factors.

After exclusions, the total sample amounted to 1,407 healthcare professionals involved in medical education. In the sample, 54.0% was female. Mean age was 45.3 years (SD 11.1), and 20.1% of healthcare professionals were specialist trainees or residents not in specialist training. In the sample, 197 healthcare professionals (14%) were working in primary care or another extramural healthcare facility, 503 (35.7%) were working in a non-university teaching hospital, and 707 (50.2%) were working in a university hospital.

### Factor analyses and reliability

For T-BNS, factor analysis on 18 items yielded 3 factors, corresponding to the items intended to measure the constructs of autonomy, competence, and relatedness. All three factors were considered reliable, with Cronbach’s alphas of 0.80, 0.86, and 0.80, respectively. In their sample of teachers working in a university hospital, Engbers et al. found Cronbach’s alphas of 0.84, 0.87 and 0.79 for the same three factors (Engbers et al., 2015).

For the subscale on contextual factors, exploratory factor analysis was performed on 13 items, searching for three factors. Three constructs (facilitators, recognition, and opportunities for teacher development) were statistically identifiable in the scale (see Appendix 1), and reliability was average to good (Cronbach’s alphas 0.62, 0.78, and 0.73 respectively).

### Feelings of autonomy, competence, and relatedness, and perceived presence of contextual factors in different healthcare contexts

Mean basic need satisfaction scores, perceived presence of contextual factors, and control variables are shown separately in Table 2 for the different healthcare contexts. The variables shown are the dependent and independent variables in the regression model used in the next section.

**Table 2:** Feelings of autonomy, competence, and relatedness, and perceived presence of contextual factors in different healthcare contexts (n = 1407)

	UH (n = 707)	DTH (n = 503)	PC (n = 197)	*p*
*Basic need satisfaction*				
Autonomy (mean (SD))	2.60 (0.62)	2.76 (0.56)	2.90 (0.53)	< 0.01*†‡
Competence (mean (SD))	2.87 (0.58)	2.74 (0.60)	2.85 (0.49)	< 0.01*
Relatedness (mean (SD))	2.68 (0.66)	2.92 (0.54)	2.75 (0.62)	< 0.01* ‡
*Contextual factors*				
Recognition (mean (SD))	2.58 (0.52)	2.70 (0.44)	2.82 (0.45)	< 0.01*†‡
Facilitators (mean (SD))	1.74 (0.84)	1.72 (0.74)	2.28 (0.78)	< 0.01 †‡
Opportunities for teacher development (mean (SD))	2.36 (0.62)	2.24 (0.60)	2.50 (0.56)	< 0.01*†‡
Role in development of education (% yes)	69.0	57.5	51.8	X^2^ 0.02
Role in coordination of education (% yes)	52.8	55.3	43.7	X^2^ < 0.01
*Control variables*				
Sex (% female)	53.0	55.9	52.8	ns
Years of experience (mean (SD))	11.0 (9.1)	11.4 (8.3)	18.1 (8.8)	< 0.01 †‡
Proportion of teaching tasks (mean % per week (SD))	20.3 (18.5)	14.6 (7.4)	24.7 (16.4)	< 0.01*†‡
Importance of education at the time of job application (% very important or quite important)	56.2	65.6	48.2	X^2^ < 0.01

*UH* university hospital, *DTH* district teaching hospital, *PC* primary care

*p*-value of ANOVA is shown, symbols show which of the comparisons differ significantly (at p = 0.05 or below). X^2^ shows p values of the chi-square test

*UH vs DTH, †UH vs PC, ‡DTH vs PC. *ns* not significant

Basic need satisfaction differed between healthcare professionals working in different contexts, with PC healthcare professionals feeling significantly more autonomous in their teaching tasks than DTH and UH healthcare professionals, and DTH healthcare professionals feeling more autonomous than UH healthcare professionals. UH healthcare professionals felt more competent than DTH healthcare professionals, while DTH healthcare professionals felt more related than healthcare professionals in other contexts.

With regard to contextual factors, PC healthcare professionals scored significantly higher on perceived presence of recognition than healthcare professionals in other contexts, and DTH healthcare professionals scored higher than UH healthcare professionals. PC healthcare professionals felt significantly better facilitated than healthcare professionals from other healthcare contexts. In addition, PC healthcare professionals perceived more opportunities for teacher development than DTH and UH healthcare professionals, while UH healthcare professionals scored significantly higher on this domain than DTH healthcare professionals. Degree of participation in development and coordination of education differed significantly between healthcare professionals in different contexts, with PC healthcare professionals being less often involved in coordination of education, and DTH and PC healthcare professionals being less often involved in development of education.

Gender did not differ significantly between groups of healthcare professionals working in different healthcare contexts. PC healthcare professionals had significantly more working experience in the healthcare profession than those in other contexts. In addition, they also spent the more time on teaching tasks than healthcare professionals from UH and DTH, while UH healthcare professionals spent more time on teaching than DTH healthcare professionals. DTH healthcare professionals more often reported that education was important to them at the time of their job application compared to other healthcare professionals, PC healthcare professionals indicated this less often.

### Relations between feelings of autonomy, competence, and relatedness, and context of employment and contextual factors

The relations between feelings of autonomy, competence, and relatedness, and context of employment and contextual factors are shown in Table 3, and explained below in more detail. In this linear regression model, we controlled for participants’ sex, years of working experience, proportion of teaching tasks in their total employment and the importance they attached to education at the time of their job application.

**Table 3:** Regression model showing relations between basic need satisfaction and contextual factors, correcting for control variables (n = 1407)

	Autonomy		Competence		Relatedness	
	B	s.e	B	s.e	B	s.e
Recognition	0.34	0.03**	0.20	0.03**	0.35	0.03**
Facilitators	0.13	0.02**	0.00	0.02	0.07	0.02**
Opportunities for teacher development	0.09	0.03**	0.11	0.03**	0.22	0.03**
Role in development of education	0.07	0.03*	0.13	0.03**	0.07	0.03*
Role in coordination of education	0.11	0.03**	0.14	0.03**	0.09	0.03**
Working in PC (1 = PC)	0.14	0.04**	-0.13	0.04**	-0.09	0.05*
Working in DTH (1 = DTH)	0.15	0.03**	-0.13	0.03**	0.25	0.03**
*Control variables*						
Sex (1 = male)	0.05	0.03	0.13	0.03**	0.03	0.03
Years of experience	0.00	0.00	0.01	0.00**	0.00	0.00
Proportion of teaching tasks (% per week)	0.00	0.00**	0.00	0.00*	0.00	0.00**
Importance of education at the time of job application	0.07	0.03*	0.14	0.03**	0.02	0.03
Intercept	1.04	0.08**	1.63	0.08**	0.92	0.08**
R2	0.29		0.24		0.31	

*s.e.* standard error, *PC* primary care, *DTH* district teaching hospital

**p < 0.01; *p < 0.05. two-tailed test

All included contextual factors significantly relate to higher feelings of autonomy in teaching tasks. Feeling appreciated (b = 0.34) as well as facilitated (b = 0.13) in teaching was associated most strongly with higher feelings of autonomy. Having opportunities for teacher development (b = 0.09) and participating in development (b = 0.07) and coordination of education (b = 0.11) was also associated with higher feelings of autonomy. Working in PC (b = 0.14) or in DTH (b = 0.15) was associated with higher feelings of autonomy.

Being appreciated (b = 0.20) as well as having opportunities for teacher development (b = 0.11) was associated most strongly with higher feelings of competence. Participating in coordination (b = 0.14) and development (b = 0.13) of education was also associated with feeling competent in teaching tasks. Working in PC (b = -0.13) and DTH (b = -0.13) was associated with lower feelings of competence. Being facilitated was not associated significantly with feelings of competence.

For relatedness, all contextual factors were associated with feeling more related in teaching tasks. Healthcare professionals who reported they felt most appreciated (b = 0.35) and had opportunities for teacher development (b = 0.22) also felt most related in teaching tasks. In addition, they more often reported they were facilitated (b = 0.07) and participated in coordination (b = 0.09) or development (b = 0.07) of education than those feeling less related. Context of employment was also of relevance, with PC healthcare professionals (b = −0.09) reporting lower feelings of relatedness, and DTH healthcare professionals (b = 0.25) reporting higher feelings of relatedness.

All control variables were positively associated with the dependent variables (Table 3). Healthcare professionals who attached more importance to teaching tasks at the time of their job application felt more autonomous and competent. Spending a larger proportion of time on teaching was associated with higher feelings of autonomy, competence, and relatedness. Male healthcare professionals reported they felt more competent in teaching tasks than female healthcare professionals, and more working experience was also associated with higher feelings of competence.

## Discussion

This study shows that basic need satisfaction in teaching, as well as perceived presence of contextual factors associated with teacher motivation, differs between healthcare contexts. Moreover, it shows how contextual factors relate to basic need satisfaction for medical teaching, independent of healthcare context. These findings are relevant as the medical educational landscape is moving towards a distributed model, in which a broad variety of new, changing, non-traditional healthcare contexts will need to take on a larger educational role.

To our best knowledge, this study is the first to explore differences in basic need satisfaction (feelings of autonomy, competence, and relatedness) in medical teaching between healthcare professionals from different healthcare contexts. Though we can only speculate, we expect that differences could be explained by factors related to differences in teaching appointments, in nature of involvement in medical education, and in organizational and professional culture. Results show that healthcare professionals working in primary care (PC) or district teaching hospitals (DTH) feel more autonomous in their teaching tasks than those working in a university hospital (UH). DTH healthcare professionals feel more related to others in teaching tasks than UH and PC healthcare professionals. These findings may reflect the autonomous nature of the work of PC healthcare professionals, possibly extending to them feeling more autonomous, but at the same time less related to others, in teaching tasks. DTH healthcare professionals on the other hand often work together in small teams or departments, who are responsible for clinical training together as a group. This may foster feelings of autonomy and relatedness in teaching as it can allow DTH healthcare professionals to shape their educational practice together.

UH healthcare professionals feel most competent in their teaching tasks, followed by PC healthcare professionals. More so than in DTH-context, medical education is viewed as one of the core tasks of a UH, which may possibly be reflected in UH healthcare professionals feeling more confident in their abilities as teachers. Similarly, this sample of PC healthcare professionals have chosen their role as dedicated PC educators (in contrast to healthcare professionals from hospital contexts), and they may have chosen their educational position because they believe in their educational competence.

In addition to exploring teachers’ basic need satisfaction in various healthcare contexts, we studied relations between contextual factors and basic need satisfaction independent of healthcare employment context. Earlier studies on motivation-related contextual factors offered non-specific insights into these relations and were situated in a single (university hospital) healthcare context. Measuring basic need satisfaction in teaching in healthcare professionals from different healthcare contexts allows us to provide a more detailed insight into how exactly different contextual factors may contribute to motivation, and how they can be used to improve teacher motivation in different contexts.

The most important contextual factor associated with teachers feeling more autonomous, competent, and related in their teaching tasks is recognition for the teacher role. This corresponds with literature showing how teacher motivation is supported by policy initiatives, organizational vision and support regarding teaching, the presence of role models, and motivated students (Bartle & Thistlethwaite, 2014; Browne et al., 2018; Dybowski & Harendza, 2014; Engbers et al., 2015; Orsini et al., 2020; Seabrook, 2003; Berg et al., 2013; Berg et al., 2015). Recognition for teaching can encourage healthcare professionals to take on teaching tasks and boost their motivation for teaching, which is especially important in the often research-focused environment of UH as well as in efficiency-driven contexts with high patient loads such as DTH and PC (Bartle & Thistlethwaite, 2014). Where earlier studies identified general relations between teacher motivation and being facilitated in terms of money, dedicated teaching time, and administrative support, we specifically found that these contextual factors mainly contribute to teachers’ feelings of autonomy, and to a lesser extent to feelings of relatedness in teaching (Browne et al., 2018; Cochran Ward et al., 2013; DaRosa et al., 2011).

We found that teacher development opportunities were associated most strongly with more feelings of relatedness, underlining the importance in the literature of involving groups of teachers in faculty development initiatives to benefit peer-learning and community formation (Barber et al., 2019; Campbell et al., 2019; de Carvalho-Filho et al., 2020), particularly because we know that faculty development initiatives aiming to form communities of teachers benefit feelings of relatedness and competence in teaching (Ten Cate et al., 2011). It might certainly be important for faculty development efforts focusing on community formation to include all healthcare professionals in a distributed educational model because some contexts, such as PC, do not easily facilitate collaboration with other teachers (Alberti & Atkinson, 2018; van Schalkwyk et al., 2020a, 2020b). The relation we found between having opportunities for teacher development and feeling more competent, furthermore, is also in line with the literature: utilizing development opportunities can boost feelings of competence, and feeling more competent, in its turn, may even lead to searching out more opportunities for further development (Dybowski & Harendza, 2014; Berg et al., 2015).

In addition, our finding that healthcare professionals who were involved in coordination and/or development of medical education reported higher basic need satisfaction is in line with findings from previous studies on teacher motivation (Blitz et al., 2018; Cochran Ward et al., 2013; Dahlstrom et al., 2005). In the current medical education landscape, UH contexts are often in the lead when it comes to medical education development. This is also visible in our sample: healthcare professionals working in UH were most often involved in development of medical education. PC healthcare professionals were least involved in coordination and development of education. We would recommend involving healthcare professionals working in other contexts more closely in developing education: not only in their own working environment, but possibly also on a more overarching level in undergraduate education or curriculum development (Blitz et al., 2018). Involving healthcare professionals in distributed learning environments more in coordination and/or development of medical education could boost their feelings of autonomy through enabling them to help shape educational practice, and boost their feelings of competence and relatedness through learning and working together with teachers in their own as well as in other contexts, with educationalists, with students, and with patients as well (Vijn et al., 2018).

Willingness to participate in curriculum development could become a mandatory topic in job interviews, particularly because we found that healthcare professionals who indicated at the time of their job interviews that teaching tasks were important show higher feelings of autonomy and competence in teaching. Interestingly, healthcare professionals working in DTH, who have the least dedicated teaching time compared to healthcare professionals in UH or PC, most often considered education to be quite or very important at the time of their job interviews. Protected time for teaching may be experienced as scarce for DTH healthcare professionals, which can form a barrier if we want to encourage healthcare professionals in distributed learning environments to be more closely involved in coordination and development of education.

In short, this study adds to literature and practice by showing how contextual factors relate to teacher motivation by satisfying basic psychological needs. These results can be utilized in a broad variety of healthcare contexts for optimal teacher basic need satisfaction, which in turn benefits medical education quality. Recognizing healthcare professionals’ teaching tasks, providing them with facilities and opportunities for developing their teaching role, and involving them in educational design and coordination allows them to develop a more autonomous motivation for medical teaching. This could be achieved, for example, by faculty development initiatives aiming to form communities of teachers, specifically including professionals from different healthcare contexts. Further, establishing connections between teachers from different contexts will hopefully allow more teachers to be involved in medical education development and coordination. In addition to boosting teacher motivation, we believe that this is an important step towards shared educational responsibilities in distributed medical education. We also recommend addressing the intention of healthcare professionals to be involved in medical education during job interviews.

A strength of this study is its timeliness: these results are relevant as well as urgent in the changing medical educational landscape. We managed to reach and include a large sample of healthcare professionals working in different contexts that participated in the study. As is common in questionnaire studies, those that have filled out the questionnaire may have done so because they feel strongly about medical education, which may have influenced the results (response bias). In this specific sample, healthcare professionals working in PC were dedicated educator healthcare professionals, which may also have influenced results towards a more positive (motivated) outcome. Because this study is cross-sectional, we cannot claim causality of the described relations between healthcare contexts and basic need satisfaction, and between contextual factors and basic need satisfaction. Additional research needs to scrutinize the causal or interacting relations further. In addition, longitudinal research is needed to determine if basic need satisfaction in medical teaching can be improved through these contextual factors. We recommend the use of qualitative research methods to shed more light on how these contextual factors can be optimized in distributed educational environments, for example to study how teachers from different healthcare contexts can learn and collaborate in faculty development programs, and in curriculum design and coordination.

## Appendix 1: Exploratory factor analysis on 13 contextual factors associated with teacher motivation

**Table:**

	Factor			Communalities
	1	2	3	
In my workplace there is enough time available for teaching tasks	**0.942**	− 0.103	0.011	0.468
In my workplace there is enough financial compensation available for teaching tasks	**0.598**	0.086	0.022	0.167
In my workplace there is enough organizational support available for teaching tasks	**0.531**	0.233	0.046	0.329
In my workplace there is enough educational support available if required	0.013	**0.732**	− 0.051	0.296
In my workplace there are career opportunities in education and training	− 0.014	**0.645**	− 0.034	0.102
In my workplace there are enough learning activities available (refresher training) for teacher self-development	0.053	**0.624**	− 0.022	0.429
In my workplace I receive feedback on my teaching tasks	0.048	**0.449**	0.126	0.814
In my workplace I am free to choose in what learning activities (refresher training) in the domain of teaching I enroll	0.011	**0.380**	0.080	0.491
When I perform teaching tasks, this is appreciated by my colleagues	0.000	− 0.078	**0.720**	0.410
When I perform teaching tasks, this is appreciated by the organization	0.107	− 0.007	**0.519**	0.186
In my workplace there are good role models in the domain of teaching	− 0.035	0.203	**0.433**	0.302
When I perform teaching tasks, this is appreciated by my patients	− 0.054	0.025	**0.420**	0.387
The learners I meet (students, trainee doctors, doctors not in specialist training, doctors in specialist training) have high motivation for learning	0.068	− 0.005	**0.285**	0.509

Bold values indicate which items belong together in the different factors

Extraction method: Maximum likelihood. rotation method: Oblimin with Kaiser normalization.
